# Supplementary material for: Expression Analysis of ZPB2a and Its Regulatory Role in Sperm-Binding in Viviparous Teleost Black Rockfish
Source: Int J Mol Sci. 2022 Aug 22;23(16):9498. doi: 10.3390/ijms23169498 (PMC9409380; doi:10.3390/ijms23169498)
Supplement: Supplementary file 1 [file ijms-23-09498-s001.zip › Table S1.pdf]

Table S1 Results of the four human ZP proteins matching to the genome of *S. schlegelii* by local Blast.

Query	Sbjct	Accession number	Identity	E value
Human_ZPB1(ZP1)	Ssc_10013305	ON685210	37%	2e-056
	Ssc_10016187	ON685211	37%	3e-050
	Ssc_10000158	NA	34%	6e-046
Human_ZPB2(ZP4)	Ssc_10013305	ON685210	38%	8e-072
	Ssc_10016187	ON685211	38%	2e-069
	Ssc_10000158	NA	36%	9e-060
Human_ZPA(ZP2)	Ssc_10013305	ON685210	33%	3e-036
	Ssc_10016187	ON685211	32%	1e-035
	Ssc_10008129	NA	30%	6e-034
Human_ZPC(ZP3)	Ssc_10014949	ON685212	41%	4e-056
	Ssc_10003055	ON685213	39%	5e-048
	Ssc_10016186	NA	38%	9e-048
Human_ZPB1-26-551*	Ssc_10013305	ON685210	38%	9e-069
	Ssc_10016187	ON685211	36%	6e-061
	Ssc_10000158	NA	35%	9e-059
Human_ZPB2-22-463*	Ssc_10013305	ON685210	39%	5e-072
	Ssc_10016187	ON685211	39%	2e-068
	Ssc_10000158	NA	36%	2e-059

* *E. coli* expressed human zona pellucida protein fragments has been proved to bind sperm *in vitro* [11, 38]
