# Supplementary material for: Expression Analysis of ZPB2a and Its Regulatory Role in Sperm-Binding in Viviparous Teleost Black Rockfish
Source: Int J Mol Sci. 2022 Aug 22;23(16):9498. doi: 10.3390/ijms23169498 (PMC9409380; doi:10.3390/ijms23169498)
Supplement: Supplementary file 1 [file ijms-23-09498-s001.zip › Table S2.pdf]

Table S2. The primers used in this study.

Gene Symbol	Primer sequence (5’-3’)	usage
**ZPB2a**	F: CGCGGATCCGTTACTGCCCAGCATCACTG	prokaryotic expression
	R: GCAAGCTTTCATCTCTGCAATTTGCCG	
**ZPB2c**	F: CTCGAGGACACTCAATATGGCCAGATT	prokaryotic expression
	R: AAGCTTTTATTGAATTCGGCTTGGGTC	
**ZPB2a**	F: GTCAGTCTGCTGGAAACAA	qPCR
	R: GTTCTCGTACACCACGTAAC	
**CRHBP**	F: TGTTTGGTGGAACTGCTATT	qPCR
	R: GTGACTCAGCACATCGTTTA	
**SPAM1**	F: CCTGCTGATCCACAGATTTC	qPCR
	R: CGTGAGAAAGACTGACACATT	
**PRM**	F: GGCAAAGAGTCCGAAGAAA	qPCR
	R: GTCGCATTCAGTGTTCATTTAG	
**IZUMO1**	F: CGTCCCGAAGCCTCAAC	qPCR
	R: CAGAGCTGTAACCGTGAGGAT	
**RPL17**	F: AGGCGACGCACCTACCG	qPCR
	R: CCTCTGGTTTGGGGACGA	
