# Supplementary figures and images for: Expression Analysis of ZPB2a and Its Regulatory Role in Sperm-Binding in Viviparous Teleost Black Rockfish
Source: Int J Mol Sci. 2022 Aug 22;23(16):9498. doi: 10.3390/ijms23169498 (PMC9409380; doi:10.3390/ijms23169498)

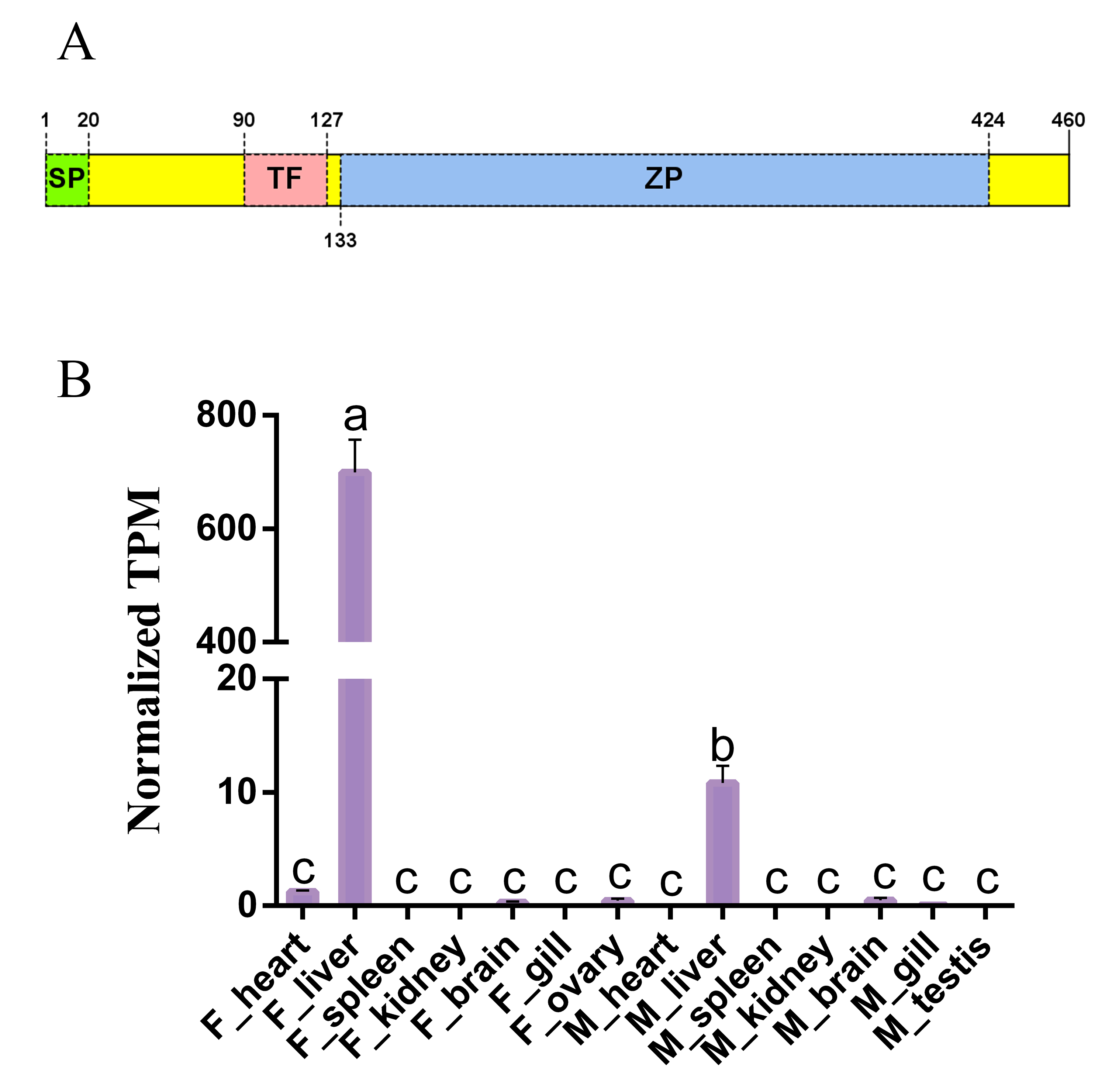

Supplement: Supplementary file 1 [file ijms-23-09498-s001.zip › Figure S1.jpg]

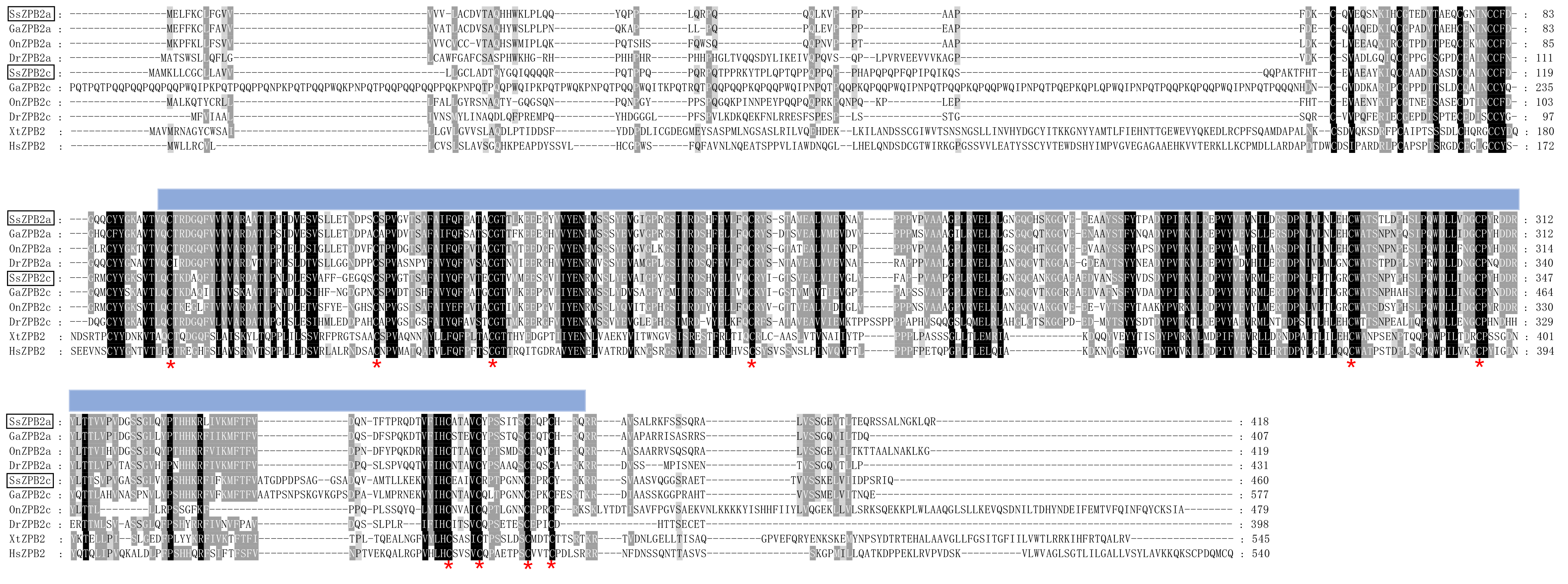

Supplement: Supplementary file 1 [file ijms-23-09498-s001.zip › Figure S2.jpg]

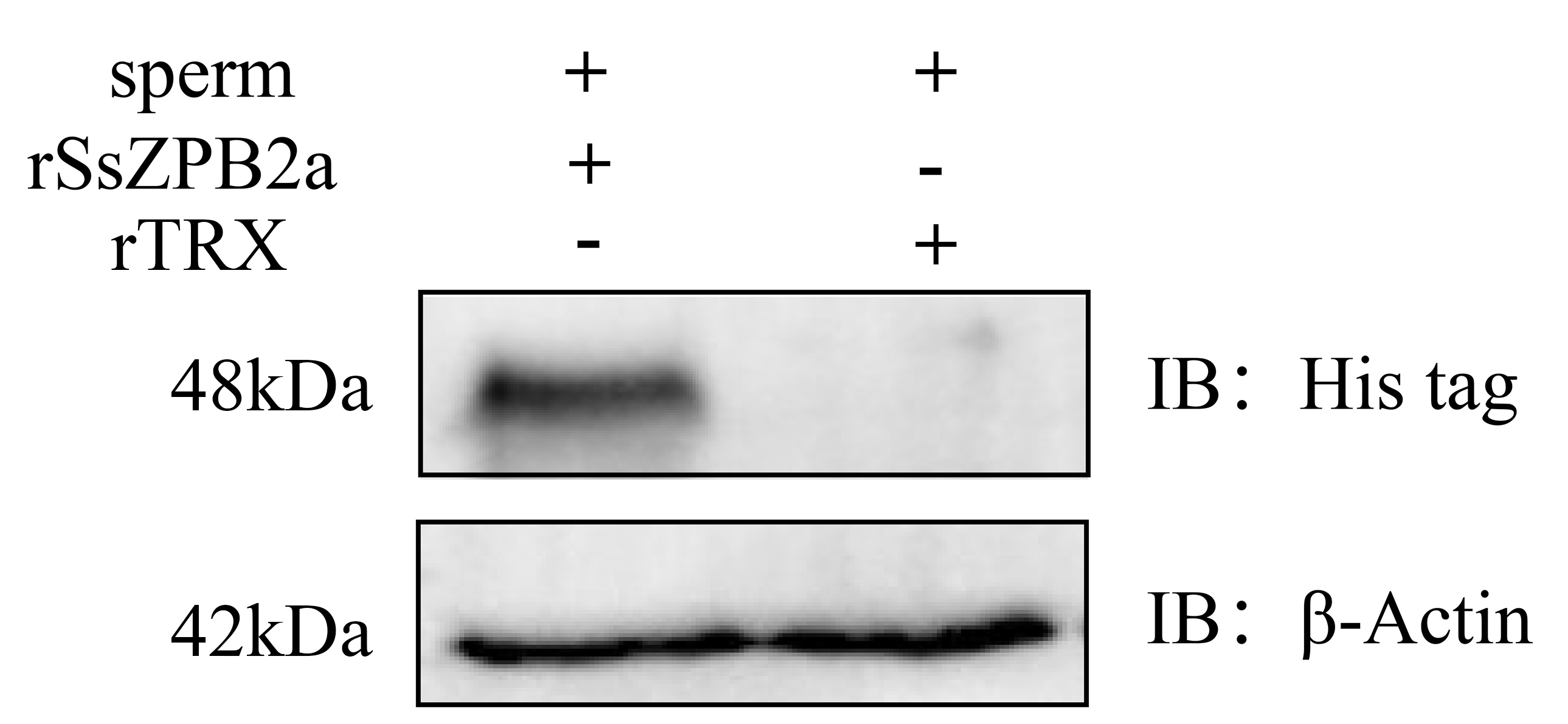

Supplement: Supplementary file 1 [file ijms-23-09498-s001.zip › Figure S3.jpg]

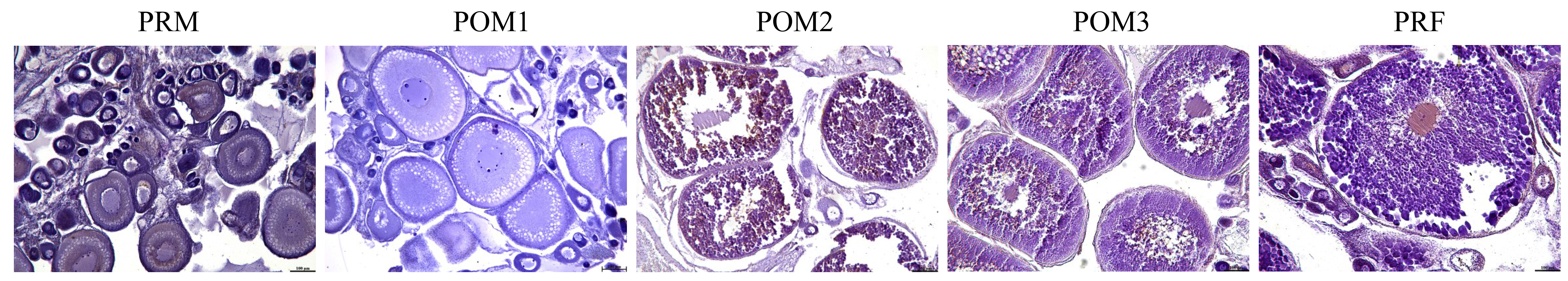

Supplement: Supplementary file 1 [file ijms-23-09498-s001.zip › Figure S4.jpg]
